# Analysis of the Cardiorespiratory Pattern of Patients Undergoing Weaning Using Artificial Intelligence

**DOI:** 10.3390/ijerph20054430

**Published:** 2023-03-01

**Authors:** Jorge Pinto, Hernando González, Carlos Arizmendi, Hernán González, Yecid Muñoz, Beatriz F. Giraldo

**Affiliations:** 1 Faculty of Engineering, Universidad Autónoma de Bucaramanga; Bucaramanga 680003, Colombia; 2 Automatic Control Department (ESAII), The Barcelona East School of Engineering (EEBE), Universitat Politècnica de Catalunya (UPC), 08019 Barcelona, Spain; 3 Institute for Bioengineering of Catalonia (IBEC), The Barcelona Institute of Science and Technology, 08019 Barcelona, Spain; 4 CIBER de Bioengeniera, Biomateriales y Nanomedicina (CIBER-BBN), 28903 Madrid, Spain

**Keywords:** mechanical ventilation, weaning, wavelet transform, neural networks

## Abstract

The optimal extubating moment is still a challenge in clinical practice. Respiratory pattern variability analysis in patients assisted through mechanical ventilation to identify this optimal moment could contribute to this process. This work proposes the analysis of this variability using several time series obtained from the respiratory flow and electrocardiogram signals, applying techniques based on artificial intelligence. 154 patients undergoing the extubating process were classified in three groups: successful group, patients who failed during weaning process, and patients who after extubating failed before 48 hours and need to reintubated. Power Spectral Density and time-frequency domain analysis were applied, computing Discrete Wavelet Transform. A new Q index was proposed to determine the most relevant parameters and the best decomposition level to discriminate between groups. Forward selection and bidirectional techniques were implemented to reduce dimensionality. Linear Discriminant Analysis and Neural Networks methods were implemented to classify these patients. The best results in terms of accuracy were, 84.61 ± 3.1% for successful versus failure groups, 86.90 ± 1.0% for successful versus reintubated groups, and 91.62 ± 4.9% comparing the failure and reintubated groups. Parameters related to Q index and Neural Networks classification presented the best performance in the classification of these patients.

## 1. Introduction

Mechanical Ventilation (MV) is a life support treatment in the Intensive Care Unit (ICU), that aims to replace the artificial form of the respiratory system. MV is becoming one of the most important scientific issues in the COVID-19 crisis because patients requiring critical care were mechanical ventilation within 24 hours of admission [1,2]. When a patient is assisted by MV, one of the main objectives of clinical practice is the recovery of spontaneous breathing in the shortest possible time. Around 40% of patients who come to an intensive care unit need mechanical ventilation [3,4], being dramatically increased with the COVID-19 [1,2]. The prolonged use of ventilatory support increases the morbidity and mortality of these patients [5,6,7], the risks of contracting other diseases, and the hospitalization cost. On the other hand, early disconnection of the mechanical ventilator, in addition to being annoying for the patient, can cause cardiopulmonary disorders. According to the literature, following the actual clinical protocols in the disconnection process, up to 25% of these patients suffer respiratory distress severe enough to have to be reintubated [3].

The weaning process of a patient can take more time than it has needed to solve the clinical problem of respiratory failure. This process becomes more difficult when respiratory assistance has been prolonged in time without the need [8]. Therefore, the study of parameters that characterize de respiratory pattern could allow estimating the optimal moment for extubation of patients, implementing mathematical models. The variability of these patterns has a non-random behaviour that can be explained by neurocentral mechanisms or by their instability in neuronal feedback loops. 

Various authors have studied this variability and possible indicators that help to select the optimal moment for the weaning of a patient [8,9,10,11,12]. The behavior of the heart rate variability is related, among others, to the action exerted by breathing on the cardiovascular system, generating a synchronization rhythm in the high-frequency range and a secondary rhythm in the low-frequency range [13]. In the response of the cardiac system, the high-frequency band is related to the activity of the parasympathetic system, associated with respiratory sinus arrhythmia. This process can be reduced with moderate or intense exercise, or with an increase in respiratory rate. The low-frequency band is related to the modulation of the sympathetic system [14]. During the extubating process, adrenal stimulation occurs, which can be resected in a fall in the parasympathetic nervous system activity, affecting the high-frequency components of the heart rhythm. The final response of the MV depends on the patient’s baseline cardiovascular conditions [15]. Several studies have been performed related to cardiorespiratory interaction and their influence on the weaning process spectral analysis of heart rate [16,17,18], respiration and blood pressure signals to study the cardiorespiratory control mechanism [19] or mutual spectral analysis of cardiac interval and respiratory flow signal [20] and the coherence between heart rate variability and respiratory flow signal [21].

Currently, the spontaneous breathing trial (SBT) is the best diagnostic test to more accurately determine whether the weaning attempt will be successful. This process needs several parameters. A single weaning parameter rarely provides sufficient accuracy to predict weaning outcomes [22]. Furthermore, this prediction decreases when the patients present with multiorgan dysfunction, advanced age, prolonged MV, and severe diseases, among others [23,24]. The lack of reliable weaning parameters is related to the heterogeneity of critically ill patients and their ever-changing clinical course [25,26,27]. The causes of weaning failure are not exclusively attributable to insufficient oxygenation or ventilation; cardiac function, volume status, muscle deconditioning, and the presence of delirium also affect weaning outcomes [28]. Most indices are based on the clinical condition recorded at a single moment, although the oxygenation, ventilatory, hemodynamic, musculoskeletal, and mental states of the patient are often unstable and vary over time.

In this study, time-frequency and statistics techniques are proposed to characterize the variability of the respiratory pattern of patients during the weaning process. Using the Wavelet decomposition method, new parameters were extracted from the time series of the electrocardiogram and respiratory flow signals. A new Q index is proposed that relates the best decomposition level and its corresponding approximation or detail coefficients of the Wavelet decomposition process. Forward selection and bidirectional search methods are implemented to reduce the dimensionality of the data. Finally, classification techniques such as neural networks and linear discriminant analysis are used to assess the best model with the best discriminant indices. This methodology allows obtaining a model, based on artificial intelligence which could contribute to the extubation process of patients considering their variability and various clinical conditions.

This paper is organized as follows: Section 2 presents the datasets used in this study; also, the set of parameters which characterizes the respiratory pattern; briefly describes the methodology, characterization techniques, and classification methods used. The classification results are presented in Section 3 and then discussed in Section 4. Finally, conclusions are presented in Section 5.

## 2. Materials and Methods

### 2.1. Database

**Figure 1:**
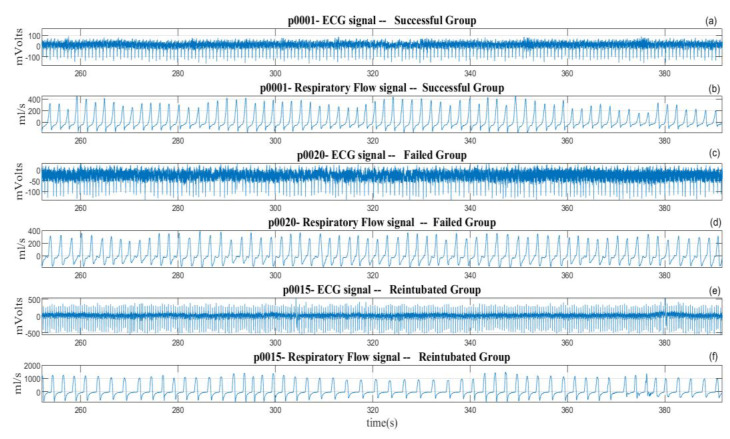
Excerpt of ECG and respiratory flow signals from patients undergoing extubation process of (**a**,**b**) a patient from the successful group, (**c**,**d**) a patient from the failed group, and (**e**,**f**) a patient from the reintubated group.

Electrocardiogram (ECG—lead II) and respiratory flow signals of 154 patients undergoing weaning from mechanical ventilation (WEANDB database) were analyzed [21]. The signals were recorded between January 2003 and April 2006, in the Intensive Care Units at the Santa Creu i Sant Pau Hospital, Barcelona, Spain, and the Getafe Hospital, Getafe, Spain. All patients were studied in accordance with the corresponding protocols, approved by each clinical research ethics committee (CEIC—HSant-Pau, and CEIC—HGetafe). The ECG signal was recorded using a SpaceLabs Medical monitor. The respiratory flow signal was obtained by a Datex-Ohmeda pneumotachograph (Validyne Model MP45-1-871 variable reluctance transducer), connected to the patient through an endotracheal tube. The signals were recorded at a sampling frequency of 250 Hz. Patients considered clinically viable for weaning underwent the T-tube test, with spontaneous breathing for 30 minutes through the endotracheal tube. According to the clinical criteria, from the weaning test, patients were classified into three groups: successful group (SG) with 94 patients (61 male, 33 female, aged: 65 ± 17 years) who were able to maintain spontaneous breathing after extubating for a minimum of 48 hours; failure group (FG) with 39 patients (24 male, 15 female, aged: 67 ± 15 years) who could not maintain spontaneous breathing during the test and were reconnected again to mechanical ventilation; and re-intubated group (RG) with 21 patients (11 male, 10 female, aged: 68 ± 14 years) that were successful in the T-tube test, but before 48 hours had to be re-intubated and reconnected to the ventilator. Figure 1 presents an excerpt of ECG and respiratory flow signals of a patient for each group in the extubating process. The respiratory flow signal present different levels of variability, according to the clinical condition of each patient. 

### 2.2. Preprocessing of Signals

**Figure 2:**
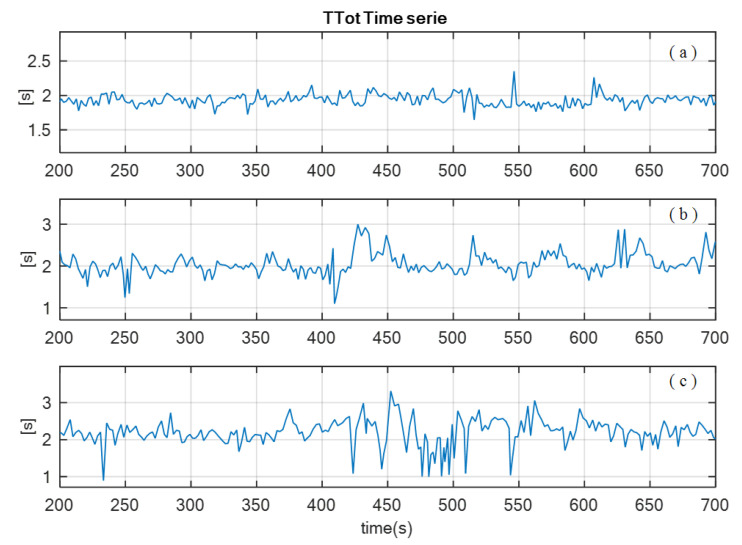
Excerpt of breathing duration time series ($T_{Tot}$) of a patient from (**a**) successful group (SG), (**b**) failed group (FG), and (**c**) reintubated group (RG).

**Figure 3:**
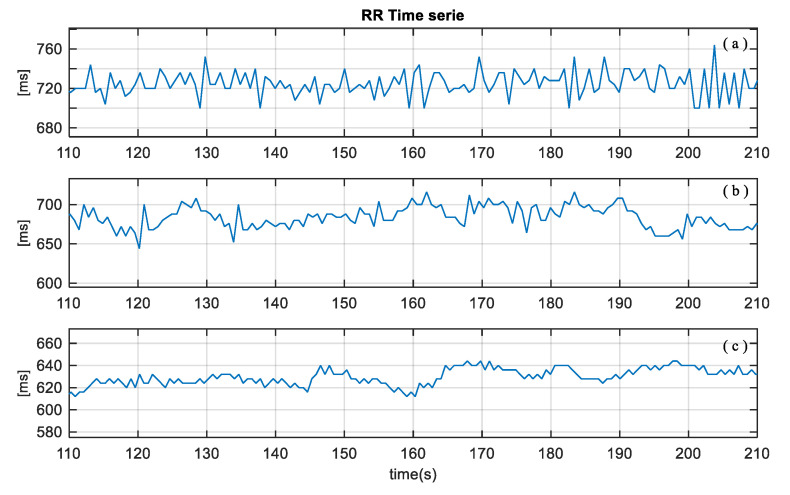
Excerpt of beat-to-beat interval (RR) from a patient of (**a**) successful group (SG), (**b**) failed group (FG), and (**c**) reintubated group (RG).

ECG and respiratory flow signals were preprocessed to reduce outliers and artifacts, and the linear trends were removed. Time series of these signals were extracted using custom algorithms, based on detection of zero crossing, and maximum and minimum values of the events of the signals. To characterize the respiratory pattern, the following time series were obtained from the respiratory flow signal: inspiratory time ($T_{I}$), expiratory time ($T_{E}$), duration of the respiratory cycle ($T_{Tot}$), tidal volume ($V_{T}$), inspiratory fraction ($T_{I}/T_{Tot}$), mean inspired flow ($V_{T}/T_{I}$), frequency-tidal volume ratio ($f/V_{T}$), where f is the respiratory rate. In addition, from the ECG signal, the cardiac beat to beat interval (RR) was obtained [15,16,17]. Figure 2 and Figure 3 illustrate an example of $T_{TOT}$ and RR time series of a patient from each group: SG, FG and RG, respectively.

To analyze the behavior of the respiratory and cardiorespiratory system at the same time, all these time series were resampled considering frequencies of 0.5 Hz, 1 Hz, 1.5 Hz, 2 Hz, 2.5 Hz and 3 Hz, applying linear interpolation. To determine the best frequency that maintains the spectrum of these new signals, the mean square error (MSE) of the power spectral density (PSD) was compared between the original and the resampled respiratory and cardiac time series. According to the results, a loss of less than 2% was obtained in the MSE at a frequency of 2 Hz for all series, so the cardiac and respiratory time series were resampled to 2 Hz.

### 2.3. Processing of the Time Series Signals

**Figure 4:**
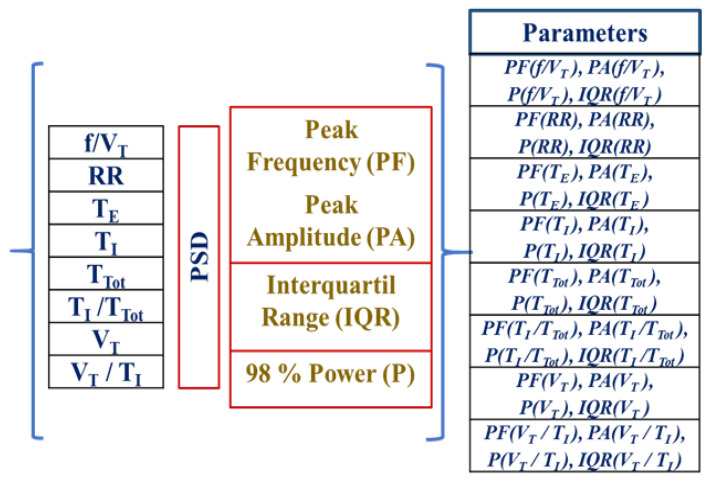
Schematic representation of the 32 variables extracted from the power spectral density for each of the 8 time series that describe the respiratory and cardiac pattern. $T_{I}$: inspiratory time, $T_{E}$ : expiratory time, $T_{Tot}$ : duration of the respiratory cycle, $V_{T}$ : tidal volume, $T_{I}/T_{Tot}$ : inspiratory fraction, $V_{T}/T_{I}$ : mean inspired flow, $f/V_{T}$ : frequency–tidal volume ratio, where f is the respiratory rate; RR: cardiac beat-to-beat interval.

To characterize the respiratory pattern and its cardiorespiratory interaction through the time series $T_{I}$, $T_{E}$, $T_{Tot}$, $V_{T}$, $T_{I}/T_{Tot}$, $V_{T}/T_{I}$, $f/V_{T}$, RR, its power spectral density (PSD) was obtained and then calculated each peak amplitude (PA), peak frequency (PF), interquartile range (IQR), and power value (P) at 98%. For each patient, 32 new variables were obtained that make up data set 1 (Dataset1). Figure 4 is a schematic representation of the analyzed dataset.

### 2.4. Wavelet Transform

The continuous Wavelet transform is given by:

(1) $$f\left(a,\tau \right)=\frac{1}{\sqrt{a}}\int_{-\infty }^{\infty }f\left(t\right)\varphi \left(\frac{t-\tau }{a}\right)\;dt$$

being a the scale factor and τ the translation in time. The scale factor $1/\sqrt{a}$ normalizes the energy. The scale a and position τ varies continuously over the real domain. For small values of a, the Wavelet is contracted over the time, generating information about the details of the signal. For high values of a, the Wavelet transform is expanded and illustrates the approximations of the signal. Thus, the scale-frequency relationship can be seen as a conglomerate of cells, where small scales correspond to high frequencies, and high scales correspond to low frequencies. The inverse relationship between time and frequency causes each of the cells to have the same area and be different from zero.

**Figure 5:**
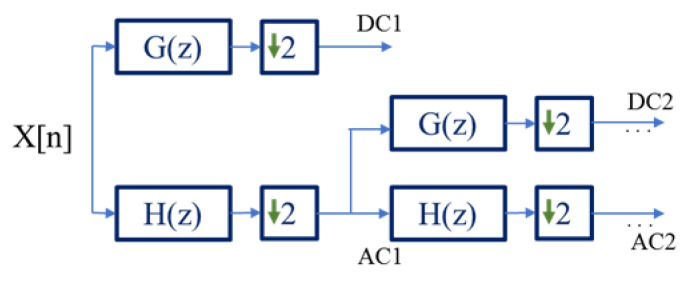
Algorithm of DWT decomposition through filter bank, where X[n] is the input signal, AC1, DC1 and AC2, DC2 represent two levels of decomposition of X[n], respectively.

The discrete Wavelet transform (DWT) allows a multiresolution analysis, applying a bank of high-pass G(z) and low-pass H(z) filters in cascade, followed by a sub-sampling stage (Figure 5). Each pair of filters represents a level of decomposition. The reconstruction of the original signal is made from a bank of synthesis filters, introducing zeros between the samples of the signal, computed with high-pass G’(z) and low-pass H’(z) filters. This reconstruction process allows the recovery of the original signal if the coefficient is not altered [20]. The approximation coefficients (AC) and detail coefficients (DC) are obtained from the decomposition stage of the signal implementing the DWT. The approximations correspond to the low frequency components of the signal, and the details with the high frequency components [20,29].

### 2.5. Extraction of Parameters

In the time-frequency analysis of the signals from the DWT, the following Wavelets families were implemented: Daubechies (1:45), Coiflets (1:5), Symlets (1:29) and of the Biorthogonal family the models 1.1, 1.3, 1.5, 2.2, 2.4, 2.6, 2.8, 3.1, 3.3, 3.5, 3.7, 3.9, 4.4, 5.5 and 6.8. In order to obtain the best mother Wavelet and level, the results of these were analyzed by comparing the MSE obtained between the original signal and the reconstructed one, implementing the maximum level of decomposition allowed by the entropy criteria, according to the methodology applied in [29]. Table 1 presents the MSE of the best Wavelets for each time series of the total of level decomposition. The family of biorthogonal Wavelets presents the smallest error for each of the analyzed data series. Implemented the corresponding Wavelets for each time series, the approximation ($AC_{j}$) and detail ($DC_{j}$) coefficients for each level of decomposition j were obtained. For each approximation and detail coefficient, the mean statistics ($\bar{X}$), standard deviation (*S*), skewness (*SK*), kurtosis (*K*) and interquartile range (*IQR*) were obtained. Finally, a total of 640 Wavelet indices that characterize the respiratory and cardiac pattern were obtained.

### 2.6. Dimensionality Reduction

To reduce the dimensionality of the data, a novel Q index based on the average values of the probability to obtain statistically significant differences (*p* < 0.05) between different parameters is proposed. The statistical significance of the parameters is obtained applying the Mann-Whitney U Test. For this study, 640 parameters obtained through the discrete wavelet transform have been analyzed. This new index relates the sum of the occurrence probability of each parameter with *p* < 0.05 with respect to its total occurrence probability and the number of elements used in the comparison. Q index is applied to approximation $AC_{j}$ and detail $DC_{j}$ coefficients of each decomposition level *j*, respectively, as follows:

(2) $$Q_{\left(AC_{j}\right)}=\frac{\sum_{i=1}^{k}(p\left(k,\;i\right)<0.05)}{\frac{1}{n_{1}n_{2}}\sum_{k=1}^{n_{1}}\sum_{i=1}^{n_{2}}p\left(k,i\right)}$$

(3) $$Q_{\left(DC_{j}\right)}=\frac{\sum_{i=1}^{k}(p\left(k,\;i\right)<0.05)}{\frac{1}{n_{1}n_{2}}\sum_{k=1}^{n_{1}}\sum_{i=1}^{n_{2}}p\left(k,i\right)}$$

being $n_{1}$ and $n_{2}$ the number of patients corresponding to each group compared, respectively. In the dimensionality reduction process, the maximum value of the Q  index is selected. This index indicates the best decomposition level and its corresponding approximation or detail coefficients of the time series computed with their corresponding statistics ($\bar{X}$, *S*, *SK*, *K* and *IQR*) values. Table 2 presents the characteristics that obtained the best value of the Q index, when comparing (1) SG vs FG, (2) SG vs RG and (3) FG vs RG, respectively. All these parameters make up data set 2 (Dataset2). 

### 2.7. Classifications Techniques

The paper compared two classification systems: neural networks (NN) and linear discriminant analysis (LDA). NN was implemented because it has demonstrated an exceptional learning capability in high dimensionality systems, even with collinear variables, allowing to adjust the parameters to avoid overfitting, improving the bias and variance of the final system. LDA is a very good contrast method as it allows the adjustment of the system hyperparameters by Bayesian methods to increase the performance avoiding system overfitting.

#### 2.7.1. Neural Networks

Neural networks are mathematical models that use learning algorithms inspired by the brain to store information. A pattern is represented by a number of features that form a vector x of dimension d within a space X ∈ Rd. A classifier maps the input space X to a finite set of classes C = {1, ..., l}. An NN is trained to execute a classification task in a set S = {(xμ, tμ), μ = 1, ..., M} using a supervised learning algorithm. The training set S consists of a vector of M features, with x μ ∈ Rd, each tagged to a class t μ ∈ C [30]. Several NN architectures and training algorithms were implemented, and the best results were obtained with a 3-hidden-layer system and one output unit. Logistic and tangential functions were implemented to activate the hidden layer and the output layer respectively. The system was trained using backpropagation method validated through the 4-fold cross-validation technique with 150 runs, this allows an accurate estimation of the error in the test data with a good balance between bias and variance, without the need to increase the computational cost. To analyze the performance of the classification, the mean accuracy of 600 trials was obtained, and to improve its generalization the Bayesian Regularization technique was implemented [30]. The feature selection was carried out applying the wrapper sequential method of elimination of characteristics forward selection and bidirectional search [31,32,33]. Due to the imbalance of the SG, FG and RG groups, the rand resampled methodology [34] was implemented to equalize the classes.

#### 2.7.2. Linear Discriminant Analysis

Let us a problem with two classes C1 and C2 and p variables, with n1 items in C1 and n2 items in C2. ${\bar{X}}_{1}$ is the average vector of the observed items in C1 and ${\bar{X}}_{2}$ is the corresponding average vector items in C2 [28]. Let ${µ}_{1}$ and ${µ}_{2}$ identify the means of the population of the predictor variables in each class and let us assume the simplification property that the covariance matrices for both classes are the same i$\left(\sum_{1}=\sum_{2}=\sum \right)$.

The linear discriminant analysis (LDA) is made explicit by assigning an item X to the $C_{1}$ if:

(4) $$D\left(x,C_{1}\right)<D\left(x,C_{2}\right)$$

where $D\left(x,C_{1}\right)={\left(x-\mu_{i}\right)}^{´}\sum^{-1}\left(x-\mu_{i}\right),$ for *i* = 1,2 results the square of the de Mahala Nobis Distance:

(5) $${\left(\mu_{1}-\mu_{2}\right)}^{´}\sum^{-1}\left[X-\frac{1}{2}\left(\mu_{1}+\mu_{2}\right)\right]>0$$

where $\mu_{i}$ can be approximated by the simple means and ∑ by the simple covariance S calculated as:

(6) $$S=\frac{\left(n_{1}-1\right)S_{1}+\left(n_{2}-1\right)S_{2}}{n_{1}-n_{2}-2}\;\;\;.$$

If $S_{1}$ y $S_{2}$ are the covariance matrix for each class, we get the Linear Discriminant Function.

(7) $${\left({\bar{X}}_{1}-{\bar{X}}_{2}\right)}^{´}S^{-1}\left[X-\frac{1}{2}\left({\bar{X}}_{1}-{\bar{X}}_{2}\right)\right]>0\;\;.$$

In the LDA, the system was trained and validated through the 4-fold cross-validation technique with 150 runs, obtaining the mean of the accuracy of 600 trials as performance measure classification. The feature selection was carried out applying the wrapper sequential method of elimination of characteristics forward selection and bidirectional search [31,32,33,35]. Due to the imbalance of the SG, FG and RG groups, the rand resampled methodology [34] was implemented to equalize the classes.

## 3. Results

In this study, the patients from the Weandb database are analyzed considering the following classification:

Successful vs failed patients (SG vs FG),
Successful vs reintubated patients (SG vs RG)
Failed vs reintubated patients (FG vs RG).

Classification by NN´s and LDA, were implemented in conjunction with forward selection and bidirectional search dimensionality reduction techniques [32,33,34,35,36] for the Dataset1 and Dataset2, separately. To perform the classification, 80% of the data were randomly chosen to find the system model, and the remaining 20% of the data were used as test data. The 80% of the data used was validated through the 4-fold cross-validation technique, where each run the groups were randomized again, generating 600 different models, obtaining the accuracy of the 150 runs implemented. The accuracy was implemented because the clinical interest of importance of the classes is equal, and it is not convenient to give more weight to one class. The multiclass training problem was not performed because of the clinical medical interest of facing the classes in a paired way, to obtain the indexes that allow us to separate the classes that clinically have a greater problem of correct classification, to perform a later study with the relevant variables of the classes faced, to give a physiological explanation and to know more about the etiology of the problem. Table 3, Table 4 and Table 5 present the parameters with the best accuracy results, in terms of mean ± standard deviation, for each comparison group. For both, Dataset1 and Dataset2, the NN´s and LDA classification methods were implemented applying forward selection and bidirectional techniques. Accuracy corresponds to 20% of the remaining data. 

From the results it is highlighted, in general, that the highest values of accuracy are achieved with the information of the dataset2, in addition the neural network algorithm presents a higher percentage than the LDA algorithm, these results are because the Q index allows to establish the coefficients of the wavelet transform that have more information, in addition, the processing of the data in the time-frequency domain allows establishing descriptors that characterize the extubating process. In this study we have obtained three reliable models for the prediction of the extubating process. With the model presented in Table 3, it can be determined if the patient should remain connected to mechanical ventilation. If they belong to the SG group, a second diagnosis can be performed with the model in Table 4, since in RG patients there may be information that the SG group does not present. 

## 4. Discussion

A novel methodology based on the calculation of the PSD and its parameters was implemented, together with the DWT in the cardiorespiratory time series. The new Q index to reduce the dimensionality of the data was applied to the parameters extracted from the DWT and their statistical-spectral. According to the results, the maximum value of Q was obtained with the statistical expressions of parameters such as common CD1-TI between SG vs FG and FG vs RG groups, $CD1-f/V_{T}$ common between SG vs RG and FG vs RG groups, and CD2-RR, $CD4-T_{E}$ and $CD3-V_{T}/T_{I}$ that are common between SG vs RG and FG vs RG groups. Furthermore, parameters related to approximation coefficients of $CA7-f/V_{T}$ and $CA1-T_{E}$, and detail coefficients of $CD2-T_{I}/T_{Tot}$, are only in SG vs FG classification group; approximation parameter $CA1-V_{T}$ is only in SG vs FG classification group; while in the FG vs RG classification group all parameters are related to the other two comparisons. Neural Network method allows modeling complex functions [30] with the Forward Selection and Bidirectional Search techniques allowing a good subset of input features to obtained. Both with the NN´s and the LDA classification techniques, a reduced number of parameters with optimal results were obtained, when classifying the three groups of patients. When comparing SG vs RG groups, the bidirectional dimensionality reduction technique presented the highest scores to classify these patients. The parameters $\bar{X}$($CD1-f/V_{T}$) and IQR($CD3-V_{T}/T_{I}$) presented the best accuracy score, being $\bar{X}\left(CD1-f/V_{T}\right)$ the most recurrent in all the bests accuracy results, comparing these groups. The bests results, depending on the accuracies, were obtained by applying the NN´s method and Wavelet transform in Dataset1: 84.61 ± 3.1% when comparing SG vs FG groups, 86.90 ± 1.0% when comparing SG vs RG groups, and 91.62 ± 4.9% when comparing FG vs RG groups. The results showed that the most relevant information to differentiate between the groups, is concentrated in the first level of the detail coefficients of the Wavelet Analysis. 

Similar studies have been done, but they differ in database and methodology. Kwong et al., [37] conducted a review of the state of the art, identifying the following databases: MIMIC-II, MIMIC-III and MIMIC-IV (publics databases), Weandb (used in this study) and other studies were based on data collected in ICU units based in UK hospitals (adult general ICU of the Sheffield Royal Hallamshire Hospital and neonatal ICU of the Royal Liverpool University Hospital). The most common machine learning algorithms are the neural network, the support vector machine (SVM) and the Adaptive Neuro Fuzzy Inference System (ANFIS); some of the ventilator variables used in these studies are inspiratory time, expiratory time, tidal volume, positive end expiratory pressure, fraction of inspired oxygen, cardiac interbeat duration (RR- interval), oxygen saturation, respiratory rate. Ossai and Wickramasinghe [38], conducted a study aimed at reviewing the efficacy of different techniques in the mechanical ventilation process using machine learning. The main parameters for the classifier design were determined with logistic regression, backward feature selection, and recursive feature elimination; successful weaning results ranged between 44% to 92%. The results of the design of three classifiers are shown in [39]: artificial neural networks, *k* nearest neighbors and support vector machine (SVM), the input variables to the system are the pressure signal, flow and ventilatory volume, during invasive mechanical ventilation of patients at Hospital San Vicente Fundación; five statistics were determined for each signal: mean, standard deviation, kurtosis, interquartile range, and skewness. The classification system can recognize respiration levels with up to 80% accuracy using the SVM algorithm. A recent study on adult ICU patients is presented in [40], the database included patient demographics, medical records, time series and respiratory events; a comparison was made between three classifiers: logistic discriminant analysis, SVM and gradient boosting method. The best classifier was SVM, which predicts extubation with an accuracy of 94.6%. As future work, it is proposed to study new techniques in the time and frequency domain of WEANDB signals to extract features that serve as input to classifiers based on the theory of convolutional neural networks. In [41], the multi-synchrosqueezing extraction transform (MSSET) method was proposed for the time-frequency analysis of electrocardiogram signals, this method allows higher accuracy in detection of peaks in the spectrum. In [42] the design of a convolutional neural network (CNN) to predict the extubation process is presented. The algorithm is validated with historical data from the MIMIC- III database. The model achieved an accuracy of 86% and an area under the receiver operating curve (AUC-ROC) of 0.94. It is important to incorporate these machine learning techniques into a real-time monitoring system that analyzes ventilatory pattern signals, seeking to reduce the burden on medical staff and clarify the MV decision scenario to which subjects undergo. The results of the classification system are similar, and in some cases, superior to those reported in the literature, with the advantage that the data required by the algorithm correspond to information on the temporal behavior of the cardiorespiratory system, information that can be recorded with biomedical equipment located in an ICU.

Regarding the clinical problem, it is of special interest to study the patients who must be reintubated during weaning process, due to their clinical implications, among others. The behavior of these patients begins by being successful and then ends by being a failure. Consequently, the diagnosis of these patients might require greater knowledge of their conditions. The results suggest the parameter ($f/V_{T}$) as a good indicator to classify the reintubated patients when compared with successful and failed groups. This result agrees with the clinical study carried out in [38], confirming its usefulness in the analysis of the physiological differences between these groups, improving the success rate in the weaning process. With this study, we suggest that detailed coefficients indices from discrete Wavelet transform can contribute to identify and classify weaning patients. We have introduced a novel model that could contribute to stratifying these patients, with the new Q index to identify the best parameters for the classification. The Q parameter can be applied to different biomedical signal classification problems that require analysis in the time-frequency domain because this index indicates the best decomposition level and its corresponding approximation or detail coefficients of the calculated time series, corresponding to the coefficients that make a greater contribution to the energy of the original signal. This study has allowed combined these techniques to describe the cardiorespiratory pattern of these patients, analyzing the interaction between 640 parameters. However, these results should be validated with a greater number of patients, especially in the reintubated group. In addition, other clinical parameters could be included in the model. Finally, the best model has been obtained with the most relevant parameters to classify each group of patients in the weaning process. One limitation of this work is related to the clinical condition and comorbidities of the patients, although there are several factors associated with weaning failure, factors such as respiratory physiological parameters, modes of mechanical ventilation, endocrine and metabolic dysfunction, ICU-acquired weakness or diaphragm dysfunction could not be fully examined in the study, because this was performed retrospectively, therefore, future prospective multicenter studies of patients undergoing SBT are necessary taking into account the factors related above.

## 5. Conclusions

Weaning can be defined as the process by which the work of breathing performed by the MV is gradually transferred to the patient, and the patient resumes spontaneous breathing. Withdrawal of respiratory support is a delicate process, which must be performed promptly to avoid indefinite patient dependence on ventilatory support, decrease morbidity and mortality, and reduce the cost of health care. The article presents the design of a classifier that can be implemented in ICUs when performing a spontaneous breathing test, providing support to the physician when establishing whether a patient can remain connected to a mechanical ventilator. Although artificial intelligence applied in these processes may present a risk of bias due to the availability of data for training the models, its implementation will facilitate the interpretation of the different variables that are recorded during an SBT process. As training data for the proposed algorithm, the information recorded during the 30-minute SBT test was used. It is recommended to carry out a study to establish the best time intervals to determine the characteristics of the respiratory cycle, which will allow establishing indicators to determine whether a patient can be disconnected from a MV. Further, these results can also be analyzed using information from the clinical condition and the correlation with these signals.

## Figures and Tables

**Table 1:** Mean squared error (MSE) of the best wavelets for each time series.

Time Series	MSE	Wavelet
f/V_T_	6 × 10^−21^	Bior 2.4
RR	1 × 10^−21^	Bior 2.6
T_E_	5 × 10^−21^	Bior 2.6
T_I_	1 × 10^−21^	Bior 2.6
T_I_/T_Tot_	1 × 10^−21^	Bior 2.4
T_Tot_	5 × 10^−21^	Bior 2.4
V_T_/T_I_	2 × 10^−21^	Bior 2.6
VT	4 × 10^−21^	Bior 2.6

**Table 2:** Parameters that obtained the maximum value of the Q index when comparing patients of each of the groups.

Comparison Group	Parameters
SG vs FG	(CA_7_-f/V_T_), (CA_8_-RR), (CA_1_-T_E_), (CD_1_-T_I_), (CD_2_-T_I_/T_Tot_), (CA_8_-T_Tot_), (CD_1_-V_T_/T_I_), (CD_7_-V_T_)
SG vs RG	(CD_1_-f/V_T_), (CD_2_-RR), (CD_4_-T_E_), (CD_4_-T_I_), (CA_7_-T_I_/T_Tot_), (CD_3_-T_Tot_), (CD_3_-V_T_/T_I_), (CA_1_-V_T_)
FG vs RG	(CD_1_-f/V_T_), (CD_2_-RR), (CD_4_-T_E_), (CD_1_-T_I_), (CA_5_-T_I_/T_Tot_), (CD_1_-T_Tot_), (CD_3_-V_T_/T_I_), (CD_8_-V_T_)

**Table 3:** Parameters with the highest accuracy (mean ± standard deviation), when comparing the groups of patients SG vs FG.

Data Base	Classification Techniques	Reduction Dimensionality Techniques	Parameters Selected	Accuracy (%)
Dataset1	NN	Forward Selection	PF(f/V_T_), P(V_T_/T_I_), IQR(T_I_/T_Tot_), PA(f/V_T_)	65.30 ± 5.9
		Bidirectional Search	PF(f/V_T_), P(V_T_/T_I_), IQR(T_I_/T_Tot_)	54.40 ± 6.9
Dataset2	NN	Forward Selection	IQR(CD1-T_I_), K(CA8-RR)	84.61 ± 3.1
		Bidirectional Search	IQR(CD1-T_I_), K(CA8-RR), IQR(CD1-V_T_/T_I_), K(CD1-T_I_), $\;\bar{X}$(CA7-f/V_T_)	80.76 ± 6.2
Dataset1	LDA	Forward Selection	PA(f/V_T_)	70.00 ± 7.8
		Bidirectional Search	PA(f/V_T_), P(f/V_T_)	70.60 ± 7.7
Dataset2	LDA	Forward Selection	IQR(CD2-T_I_/T_Tot_)	71.64 ± 0.8
		Bidirectional Search	IQR(CD2-T_I_/T_Tot_)$\;\bar{X}$(CA7-f/V_T_), IQR(CD1-V_T_/T_I_)	72.13 ± 4.8

**Table 4:** Parameters with the highest accuracy (mean ± standard deviation), when comparing the groups of patients SG vs RG.

Data Base	Classification Techniques	Reduction Dimensionality Techniques	Parameters Selected	Accuracy (%)
Dataset1	NN	Forward Selection	P(RR)	78.23 ± 0.7
		Bidirectional Search	P(RR), PF(f/V_T_)	86.82 ± 0.7
Dataset2	NN	Forward Selection	$\bar{X}$(CD1-f/V_T_)	82.60 ± 3.0
		Bidirectional Search	$\bar{X}$(CD1-f/V_T_), IQR(CD3-V_T_/T_I_)	86.90 ± 1.0
Dataset1	LDA	Forward Selection	PF(f/V_T_)	80.91 ± 6.4
		Bidirectional Search	PF(f/V_T_), IQR(V_T_/T_I_)	81.33 ± 6.8
Dataset2	LDA	Forward Selection	$\bar{X}$(CD1-f/V_T_)	83.33 ± 7.4
		Bidirectional Search	$\bar{X}$(CD1-f/V_T_), IQR(CD3-T_Tot_)	82.55 ± 7.5

**Table 5:** Parameters with the highest accuracy (mean ± standard deviation), when comparing the groups of patients FG vs RG.

Data Base	Classification Techniques	Reduction Dimensionality Techniques	Parameters Selected	Accuracy (%)
Dataset1	NN	Forward Selection	PF(f/V_T_), PA(T_I_/T_Tot_), PA(T_I_), PA(RR), PA(f/V_T_), IQR(T_Tot_), IQR(CD1-T_I_), IQR(RR), IQR(f/V_T_)	50.00 ± 1.6
		Bidirectional Search	PF(f/V_T_), PF(T_I_/T_Tot_), PA(T_I_), PA(RR), PA(f/V_T_), P(T_Tot_)	58.36 ± 1.3
Dataset2	NN	Forward Selection	S(CD1-T_Tot_), $\;\bar{X}$(CD1-f/V_T_), IQR(CD1-f/V_T_), K(CD2-RR), Sk(CD1-f/V_T_), K(CD1-f/V_T_)	83.31 ± 3.4
		Bidirectional Search	S(CD1-T_Tot_),$\;\bar{X}$(CD1-f/V_T_), IQR(CD1-f/V_T_), K(CD2-RR), Sk(CD1-f/V_T_), K(CD1-f/V_T_), S(CD1-f/V_T_), $\bar{X}$(CA5-T_I_/T_Tot_), S(CD1-T_I_), K(CD1-T_Tot_), Sk(CD1-T_Tot_), IQR(CD2-RR), K(CD4-T_E_)	91.62 ± 4.9
Dataset1	LDA	Forward Selection	PF(f/V_T_), PA(f/V_T_), PA(T_E_), PA(T_I_/T_Tot_), P(f/V_T_)	76.00 ± 10.8
		Bidirectional Search	$\bar{X}$(CD2-RR), S(CD2-RR)	75.34 ± 11.3
Dataset2	LDA	Forward Selection	PA(f/V_T_), IQR(T_Tot_)	75.36 ± 11.3
		Bidirectional Search	$\bar{X}$(CD2-RR)	80.15 ± 10.6

## Data Availability

The data that support the findings of this study are available from Dr. Beatriz F. Giraldo, but restrictions apply to the availability of these data, which were used under license for the current study, and so are not publicly available. The dataset is not publicly available due to the conditions that were set when the protocol was defined, and the patients were recorded.

## References

[1] Ziehr D.R., Alladina J., Petri C.R., Maley J.H., Moskowitz A., Medoff B.D., Hibbert K.A., Thompson B.T., Hardin C.C. (2020). Respiratory pathophysiology of mechanically ventilated patients with COVID-19: A cohort study. Am. J. Respir. Crit. Care Med..

[2] Mahase E. (2020). COVID-19: Most patients require mechanical ventilation in the first 24 hours of critical care. Br. Med. J..

[3] Tobin M.J. (2004). Of principles and protocols and weaning. J. Respir. Crit. Care Med..

[4] Magalhães P.A., Camillo C.A., Langer D., Andrade L.B., do Carmo M., Gosselink R. (2018). Weaning failure and respiratory muscle function: What has been done and what can be improved?. Respir. Med..

[5] Girault C., Daudenthun I., Chevron V., Tamion F., Leroy J., Bonmarchand G. (1999). Noninvasive ventilation as a systematic extubation and weaning technique in acute-on-chronic respiratory failure: A prospective, randomized controlled study. Am. J. Respir. Crit. Care Med..

[6] Hsu J.-C., Chen Y.-F., Lin H.-H., Li C.-H., Jiang X. (2007). Construction of prediction module for successful ventilator weaning. Proceedings of the International Conference on Industrial, Engineering and Other Applications of Applied Intelligent Systems.

[7] Randolph A.G., Wypij D., Venkataraman S.T., Hanson J.H., Gedeit R.G., Meert K.L., Luckett P.M., Forbes P., Lilley M., Thompson J. (2002). Effect of mechanical ventilator weaning protocols on respiratory outcomes in infants and children: A randomized controlled trial. JAMA.

[8] Casaseca-de-la-Higuera P., Martín Fernández M., Alberola López C. (2006). Weaning from mechanical ventilation: A retrospective analysis leading to a multimodal perspective. IEEE Trans. Biomed. Eng..

[9] Benchetrit G. (2000). Breathing pattern in humans: Diversity and individuality. Respir. Physiol..

[10] Caminal P., Dominge L., Giraldo B., Vallverdú M., Benito S., Vázquez G., Kaplan D. (2004). Variability analysis of the respiratory volume based on non-linear prediction methods. Med. Biol. Eng. Comput..

[11] Bruce E.N. (1996). Measures of respiratory pattern variability. Bioengineering Approaches to Pulmonary Physiology and Medicine.

[12] Orini M., Giraldo B.F., Bailón R., Vallverdú M., Mainardi L., Benito S., Diaz I., Caminal P. (2008). Time-frequency analysis of cardiac and respiratory parameters for the prediction of ventilator weaning. Proceedings of the 2008 30th Annual International Conference of the IEEE Engineering in Medicine and Biology Society.

[13] Shen H.-N., Lin L.-Y., Chen K.-Y., Kuo P.-H., Yu C.-J., Wu H.-D., Yang P.-C. (2003). Changes of heart rate variability during ventilator weaning. Chest.

[14] Pinsky M.R. (2005). Cardiovascular issues in respiratory care. Chest.

[15] Trapero J., Arizmendi C., Forero C., Lopez S., Giraldo B. (2017). Cardiorespiratory interaction using nonlinear data processing techniques in patients undergoing test tube t. Proceedings of the VII Latin American Congress on Biomedical Engineering CLAIB 2016.

[16] Trapero J., Arizmendi C., González H., Forero C., Giraldo B.F. (2017). Nonlinear dynamic analysis of the cardiorespiratory system in patients undergoing the weaning process. Proceedings of the 2017 39th Annual International Conference of the IEEE Engineering in Medicine and Biology Society (EMBC).

[17] Arizmendi C., Solano E., Gonzalez H., Acuña H.G., Giraldo B. (2018). Analysis of cardiorespiratory interaction in patients submitted to the t-tube test in the weaning process implementing symbolic dynamics and neural networks. Proceedings of the 2018 International Conference on Artificial Intelligence and Big Data (ICAIBD).

[18] Mainardi L.T. (2009). On the quantification of heart rate variability spectral parameters using time–frequency and time-varying methods. Philos. Trans. R. Soc. A Math. Phys. Eng. Sci..

[19] Arcentales A., Giraldo B.F., Caminal P., Diaz I., Benito S. (2010). Spectral analysis of the RR series and the respiratory flow signal on patients in weaning process. Proceedings of the 2010 Annual International Conference of the IEEE Engineering in Medicine and Biology.

[20] Mallat S. (1999). A Wavelet Tour of Signal Processing.

[21] Arcentales A., Caminal P., Diaz I., Benito S., Giraldo B. (2015). Classification of patients undergoing weaning from mechanical ventilation using the coherence between heart rate variability and respiratory flow signal. Physiol. Meas..

[22] Heunks L.M., van der Hoeven J.G. (2010). Clinical review: The ABC of weaning failure-a structured approach. Crit. Care.

[23] Demiralp B., Koenig L., Xu J., Soltoff S., Votto J. (2021). Time spent in prior hospital stay and outcomes for ventilator patients in long-term acute care hospitals. BMC Pulm. Med..

[24] Villalba D., Rossetti G.G., Scrigna M., Colling J., Rocco A., Matesa A., Areas L., Golfarini N., Pini P., Hannun M. (2020). Prevalence of and risk factors for mechanical ventilation reinstitution in patients weaned from prolonged mechanical ventilation. Respir. Care.

[25] Videtta W., Vallejos J., Roda G., Collazos H., Naccarelli N., Tamayo A., Calderón N., Bairaclioti A., Yoshida M., Vandaele G. (2021). Predictors of successful extubation in neurocritical care patients. Intracranial Press. Neuromonit..

[26] Baptistella A.R., Mantelli L.M., Matte L., Carvalho M.E.d.R.U., Fortunatti J.A., Costa I.Z., Haro F.G., Turkot V.L.d.O., Baptistella S.F., de Carvalho D. (2021). Prediction of extubation outcome in mechanically ventilated patients: Development and validation of the Extubation Predictive Score (ExPreS). PLoS ONE.

[27] Leonov Y., Kisil I., Perlov A., Stoichev V., Ginzburg Y., Nazarenko A., Gimelfarb Y. (2020). Predictors of successful weaning in patients requiring extremely prolonged mechanical ventilation. Adv. Respir. Med..

[28] Fontela P.C., Glaeser S.S., Martins L.F., Condessa R.L., Prediger D.T., Forgiarini S.G., Forgiarini L.A., Lisboa T.C., Friedman G. (2021). Medical research council scale predicts spontaneous breathing trial failure and difficult or prolonged weaning of critically ill individuals. Respir. Care.

[29] Guo D.-f., Zhu W.-H., Gao Z.-M., Zhang J.-q. (2000). A study of wavelet thresholding denoising. 2000—ICSP 2000, 2000 5th International Conference on Signal Processing Proceedings, Proceedings of the 16th World Computer Congress 2000, Beijing, China, 21–25 August 2000.

[30] Foresee F.D., Hagan M.T. (1997). Gauss-Newton Approximation to Bayesian Learning. Proceedings of the International Conference on Neural Networks (ICNN’97).

[31] Arizmendi C., Vellido A., Romero E. (2012). Classification of human brain tumours from mrs data using discrete wavelet transform and bayesian neural networks. Expert Syst. Appl..

[32] Kohavi R., John G.H. (1997). Wrappers for feature subset selection. Artif. Intell..

[33] Arizmendi C., Vellido A., Romero E. (2012). Preprocessing mrs information for classification of human brain tumours. Medical Applications of Intelligent Data Analysis: Research Advancements.

[34] Japkowicz N. The class imbalance problem: Significance and strategies. Proceedings of the MICAI 2000: Advances in Artificial Intelligence: Mexican International Conference on Artificial Intelligence.

[35] Mika S., Ratsch G., Weston J., Scholkopf B., Mullers K.-R. (1999). Fisher discriminant analysis with kernels. Neural Networks for Signal Processing IX: Proceedings of the 1999 IEEE Signal Processing Society Workshop (cat. No. 98th8468), Madison, WI, USA, 25 August 1999.

[36] Liu H., Yu L. (2005). Toward integrating feature selection algorithms for classification and clustering. IEEE Trans. Knowl. Data Eng..

[37] Kwong M.T., Colopy G.W., Weber A.M., Ercole A., Bergmann J.H. (2019). The efficacy and effectiveness of machine learning for weaning in mechanically ventilated patients at the intensive care unit: A systematic review. Bio-Des. Manuf..

[38] Ossai C.I., Wickramasinghe N. (2021). Intelligent decision support with machine learning for efficient management of mechanical ventilation in the intensive care unit–a critical overview. Int. J. Med. Inform..

[39] Castro L.F.B., Santacruz L.F.E., Sánchez M.B.S. (2020). Work of breathing estimation during spontaneous breathing test using machine learning techniques. Proceedings of the 2020 IEEE Colombian Conference on Applications of Computational Intelligence (IEEE ColCACI 2020).

[40] Fabregat A., Magret M., Ferré J.A., Vernet A., Guasch N., Rodríguez A., Gómez J., Bodí M. (2021). A machine learning decision-making tool for extubation in intensive care unit patients. Comput. Methods Programs Biomed..

[41] Wang Y., Zhou W., Zhao X., Chen C., Chen W. (2021). Msset: A high-performance time-frequency analysis method for sparse-spectrum biomedical signal. Comput. Biol. Med..

[42] Jia Y., Kaul C., Lawton T., Murray-Smith R., Habli I. (2021). Prediction of weaning from mechanical ventilation using convolutional neural networks. Artif. Intell. Med..

